# Comparative Analysis of Microbiome Metagenomics in Reintroduced Wild Horses and Resident Asiatic Wild Asses in the Gobi Desert Steppe

**DOI:** 10.3390/microorganisms10061166

**Published:** 2022-06-07

**Authors:** Liping Tang, Yunyun Gao, Liping Yan, Huiping Jia, Hongjun Chu, Xinping Ma, Lun He, Xiaoting Wang, Kai Li, Defu Hu, Dong Zhang

**Affiliations:** 1 School of Ecology and Nature Conservation, Beijing Forestry University, 35 Tsinghua East Road, Beijing 100083, China; tanglp@bjfu.edu.cn (L.T.); en130434@bjfu.edu.cn (Y.G.); yanlp@bjfu.edu.cn (L.Y.); jiahuiping@bjfu.edu.cn (H.J.); likai_sino@sina.com (K.L.); hudf@bjfu.edu.cn (D.H.); 2 Institute of Forestry Ecology, Xinjiang Academy of Forestry Sciences, Urumqi 830002, China; hongjunchu@vip.163.com; 3 Xinjiang Kalamaili Mountain Ungulate Nature Reserve Management Center, Urumqi 830000, China; mxp6928188@163.com; 4 China Wildlife Conservation Association, Beijing 100714, China; henry714@foxmail.com (L.H.); xiaotingwang65@sohu.com (X.W.)

**Keywords:** Przewalski’s horses, Asiatic wild asses, sympatric equids, metagenomics, microbiome

## Abstract

The gut microbiome offers important ecological benefits to the host; however, our understanding of the functional microbiome in relation to wildlife adaptation, especially for translocated endangered species, is lagging. In this study, we adopted a comparative metagenomics approach to test whether the microbiome diverges for translocated and resident species with different adaptive potentials. The composition and function of the microbiome of sympatric Przewalski’s horses and Asiatic wild asses in desert steppe were compared for the first time using the metagenomic shotgun sequencing approach. We identified a significant difference in microbiome composition regarding the microbes present and their relative abundances, while the diversity of microbe species was similar. Furthermore, the functional profile seemed to converge between the two hosts, with genes related to core metabolism function tending to be more abundant in wild asses. Our results indicate that sympatric wild equids differ in their microbial composition while harboring a stable microbial functional core, which may enable them to survive in challenging habitats. A higher abundance of beneficial taxa, such as *Akkermansia*, and genes related to metabolism pathways and enzymes, such as lignin degradation, may contribute to more diverse diet choices and larger home ranges of wild asses.

## 1. Introduction

In the context of global climate change and human activity, most animals rely on their phenotypic plasticity, which is more mutable than the genome, to adapt to new environments and avoid extinction. Gut microorganisms have been shown to have an important impact on many aspects of host health, including immunity [1,2], metabolism [3,4], and behavior [5]. The recently introduced “hologenome” or “extended genome” concept proposes that individual organisms should be considered along with their microbiome to function as a biological entity of selection [6,7].

Microbiome plasticity may influence host phenotypes and allow the host to adapt to rapidly changing environments [8]. However, research on the impact of the gut metagenome on the host phenome is in its initial stage [8], and the impact in ecological and evolutionary frameworks under natural conditions is yet to be assessed [9]. This knowledge is especially important for translocated populations exposed to dramatic environmental changes, such as reintroduced animals and some invasive species [10]. Studying these contexts can provide insight into animal conservation and invasive species control, as microbial engineering is a promising way to achieve ecological balance by increasing or decreasing the ecological adaptability of animals.

The reintroduced Przewalski’s horse (*Equus ferus przewalskii*) and native Mongolian wild ass (*Equus hemionus hemionus*) distributed in the Junggar Basin provide a good model for studying microbiome divergence between hosts with different adaptive plasticities. A total of 24 Przewalski’s horses from Germany, the United Kingdom, and the United States were reintroduced in China in 1985 for captive breeding, and 27 individuals from breeding groups were released into Kalamaili Mountain Ungulate Nature Reserve (KNR) in 2001 [11]. The free-ranging Przewalski’s horse population in KNR has increased and is estimated to have over 200 individuals [12]. The current population of resident Mongolian wild asses in the same reserve is estimated to be over 3000 individuals [13]. Przewalski’s horses are classified as “Endangered” species, while Asiatic wild asses are classified as “Near Threatened” on the IUCN red list [14].

As the last representative of wild horses on the planet, Przewalski’s horses were considered to be worse adapted to arid conditions than Asiatic wild asses [15,16]. Both wild horses and wild asses are grazing herbivores, consuming grasses (e.g., *Stipa* spp.), forbs (e.g., *Salsola* spp.), and shrubs (e.g., *Haloxylon*), with highly overlapping dietary niches [17,18]. However, wild asses are highly seasonal and ate more shrubs in autumn and winter, while Przewalski’s horses are consistently adapted to more productive habitat types, are less versatile in their feeding choices, and are more constrained by water availability [18,19,20]. In marginal habitats, such as the Junggar Basin, where water and food resources are scarce, ecological niche separation is crucial for sustaining the coexistence of sympatric species. Equids are hindgut fermenters that rely on fermentation by intestinal microorganisms to digest diets with high fiber and insoluble carbohydrates [21]. Intestinal microbiota can help expand their dietary options, thus optimizing allocation strategies for animals in the same region.

Metagenomics, in which all DNA fragments in a sample are sequenced rather than only amplicons that target specific genomic regions [22], has emerged as an effective tool for investigating the diversity and functional potential of the microbiome [23]. This technique offers an avenue to understand how the microbiome affects the health and persistence of wildlife animals [24]. To the best of our knowledge, only limited metagenomic analyses of the functional aspects of equid gut microbiota have been reported [25,26].

In this study, we assessed and compared the composition and functional potential of sympatric equid microbiomes distributed in the Desert steppe using metagenomic sequencing. At present, the Junggar Gobi is the only place where wild horses and wild asses coexist, which provides a valuable opportunity to investigate whether gut microbial communities have diverged in their flexibility between wild equids under the same natural conditions. We hypothesized that reintroduced wild horses harbor fewer bacteria and genes related to cellulose degradation and energy metabolism than wild asses.

## 2. Materials and Methods

### 2.1. Sample Collection

**Figure 1:**
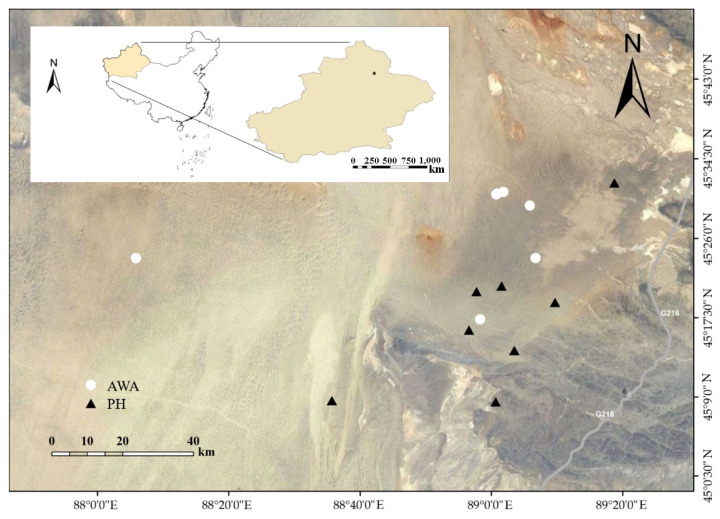
Locations of the sampling site for Przewalski’s horse (PH, dark triangles) and Asiatic wild asses (AWA, white dots) in Kalamaili Mountain Ungulate Nature Reserve.

Fresh fecal samples from eight adult wild horses and eight wild asses were collected from the KNR (44°36′–46°00′ N, 88°30′–90°03′ E) in Xinjiang, China in August, 2019 (Figure 1). During the sampling period, wild horses were closely observed while wild asses could only be observed far away due to a long alert distance, hence individual information for wild asses was not recorded. Feces were collected in sterile centrifuge tubes within 10 min of defecation. Samples were labelled and stored in a mobile refrigerator, transported to our laboratory, and stored at −20 °C until DNA extraction. The protocols for the present study were approved by the Ethics and Animal Welfare Committee of Beijing Forestry University (EAWC_BJF_2021012), and the study was conducted in accordance with approved guidelines.

### 2.2. DNA Extraction and Metagenomic Sequencing

DNA extraction and metagenomic sequencing were performed as previously described with slight modifications [27]. Briefly, DNA was isolated using the E.Z.N.A Soil DNA Kit (Omega Bio-tek, Norcross, GA, USA). Genomic DNA samples were randomly fragmented using a Covaris M220 (Gene Company Limited, Hong Kong, China) to 400 bp in size for library construction. After filtering using a NEXTFLEX Rapid DNA-Seq kit (Bio Scientific, Austin, TX, USA), paired-end sequencing was performed using an Illumina Novaseq 6000 platform (Illumina Inc., San Diego, CA, USA) by MajorBio-Pharm Technology Co. Ltd. (Shanghai, China). All sequence data obtained were deposited in the NCBI Sequence Read Archive (accession number: PRJNA814825).

### 2.3. Bioinformatics

Raw sequencing reads obtained from the Illumina Novaseq sequencing platform were processed using Trimmomatic software [28]. In this step, low quality reads were removed (including reads containing adapters, reads with more than 10% unknown bases, and reads with over 50% low Q-value (≤10) bases). Sequences in each sample were individually assembled using MEGAHIT 1.0 [29] and used for subsequent analyses. Open reading frames (ORFs) of assembled contigs with lengths over 800 bp were predicted using Prodigal software [30]. The contigs were then translated into amino acid sequences. To construct non-redundant gene catalogues, all predicted genes with 95% identity (90% coverage) were clustered using CD-HIT software, and the longest sequences of each cluster were used as a representative gene catalogue [31]. To obtain the relative abundance of each gene, Bowtie2 was used to compare the high-quality reads from each sample against the representative catalogue (identity > 95%) [32]. DIAMOND was employed for the taxonomic and functional annotation of unigenes. For taxonomic annotation, genes were aligned with prokaryote, archaea, eukaryote, and virus sequences extracted from the NCBI NR database (version: 2021.11) using blastp (e-value ≤ 1 × 10^−5^) [33]. The lowest common ancestor (LCA) algorithm was employed in MEGAN [34] to obtain a taxonomic profile. For microbiome functional profiling, the non-redundant gene set was searched against the Kyoto Encyclopedia of Genes and Genomes (KEGG) database [35] and the Carbohydrate-Active enzymes (CAZy) database [36] using DIAMOND software [33]. The match result with the highest score (one HSP > 60 bits) was selected for subsequent analysis.

For statistical analyses, minimum read filtering was applied by MicrobiomeAnalyst [37] for alpha and beta diversity calculations. Differences in the bacterial taxonomic composition between wild horses and wild asses were evaluated by non-metric multidimensional scaling (NMDS) using weighted and unweighted UniFrac indices. Analysis of similarities (ANOSIM) was conducted to determine the statistical differences between hosts using the vegan package in R 4.0.2. The linear discriminant effect size (LEfSe) with the Kruskal–Wallis test was applied to identify biomarkers with significant abundance differences between groups [38]. Linear discriminant analysis (LDA) was used to estimate the effect size of each differentially abundant trait. The Kruskal–Wallis test with Tukey–Kramer post hoc test to identify significantly different metabolic pathways employed by the microbial communities between groups was performed using the STAMP [39]. Further, we used Metastats [40] software to identify CAZymes families that were in significantly different gene abundances and the P values were adjusted for false discovery rate of 0.05.

## 3. Results

### 3.1. Sequencing Quality and Microbial Diversity

Metagenomic sequencing of fecal samples from eight wild horses and eight wild asses yielded approximately 79 to 93 million reads per sample. After removing short and low-quality sequences (see Section 2.3), approximately 2% of the total reads remained for subsequent analyses (Table S1). A total of 132.94 million clean reads (average ± SD = 83.09 ± 3.82) were generated and subsequently assembled into 11.67 million contigs (average ± SD = 0.73 ± 0.05), with contig lengths ranging from 500 to 616,284 bp (Table S2). A total of 9,922,959 unigenes were predicted based on contigs, ranging from 201 to 35,655 bp in length (Table S2), of which 177,618 unigenes were shared by all samples. These unigenes were used for further downstream taxonomic analysis. Approximately 96–99% of the unigenes were classified as bacterial genes. Archaea, eukaryotes, and viruses showed lower relative abundance (<3%). In total, 146 bacterial phyla, 228 classes, 373 orders, 708 families, 2692 genera, and 14,125 species were identified. Rarefaction curves showed that the sequences covered almost all bacterial species in the fecal samples (Figure S1).

**Figure 2:**
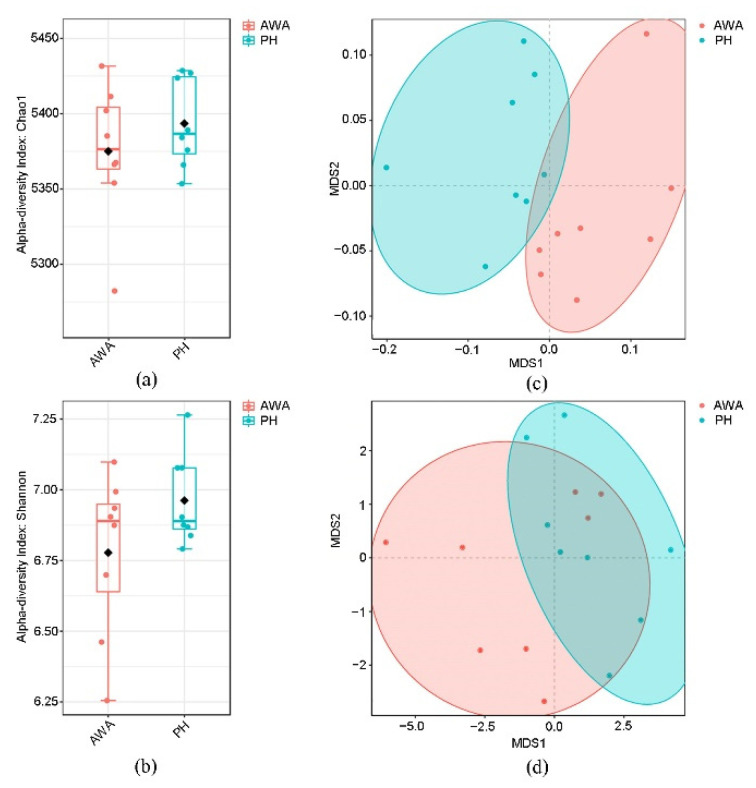
The intestinal microbial alpha-diversity and community composition in Przewalski’s horses (PH) and Asiatic wild asses (AWA). (**a**,**b**) Box plots showing; (**a**) the Chao index; and (**b**) the Shannon index of microbial species in the samples. (**c**,**d**) NMDS analysis showing the microbial community composition based on; (**c**) weighted UniFrac; and (**d**) unweighted UniFrac distance. Stress values lower than 0.2 indicate that the result is reliable.

Bacterial diversity and richness were evaluated using the Shannon index and the Chao estimator, respectively. Although both indices were higher in wild horses than wild asses, the differences were not statistically significant (Figure 2a,b: Shannon, *p* = 0.51; Chao, *p* = 0.65).

### 3.2. Differences in Intestinal Microbial Community Structure

**Figure 3:**
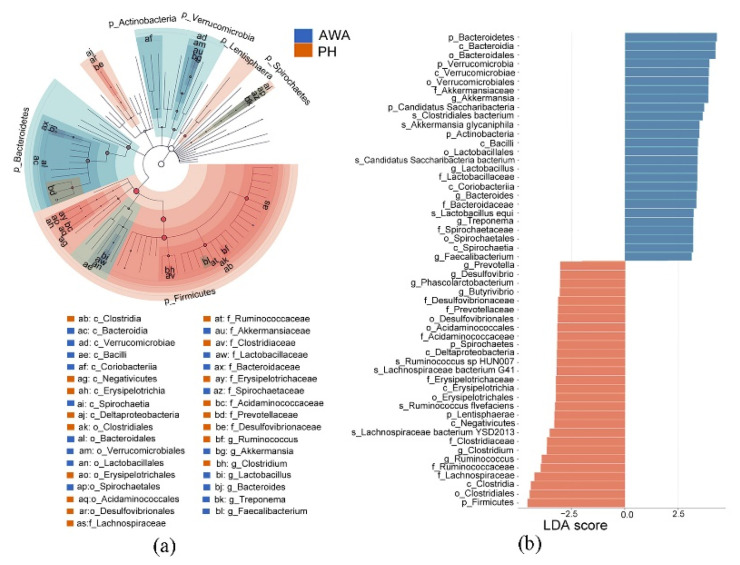
LefSe analysis. (**a**) Cladogram showing the microbial taxa with significant differences in abundance between Przewalski’s horses (PH) and Asiatic wild asses (AWA). Taxonomic hierarchies were arranged from the inside to the outside (from genus to phylum) in the cladogram; (**b**) Taxa with significant differences that have an LDA score > the threshold value of 3. Red and blue nodes and bars represent differentially abundant taxa between groups (red = more enriched in PH, blue = enriched in AWA).

The most dominant bacterial phyla in both wild horses and wild asses were Firmicutes (64–79%), followed by Bacteroidetes (6–24%) and Verrucomicrobia (2–13%) (Figure S2a). The most frequently identified genera were *Ruminococcus*, *Clostridium*, *Akkermansia*, and *Prevotella* (Figure S2b). The NMDS plot revealed significant differences in the intestinal bacterial community at the species level between wild horses and wild asses based on weighted UniFrac distance (ANOSIM: *p* < 0.01, R = 0.361; Figure 2c). However, there is an overlap between the two groups based on the unweighted UniFrac distance (Figure 2d). Biomarkers that were differently expressed between wild horses and wild asses were identified using the Lefse analysis (LDA score > 3). Wild horses had a higher proportion of Firmicutes, Lentisphaerae, and Spirochaetes phyla, and a decreased abundance of Bacteroidetes, Verrucomicrobia, Candidatus Saccharibacteria, and Actinobacteria (Figure 3). At the family and genus levels, Ruminococcaceae (*Ruminococcus*), Clostridiaceae (*Clostridium*), Erysipelotrichaceae, Acidaminococcaceae (*Phascolarctobacterium*), Prevotellaceae (*Prevotella*), and Desulfovibrionaceae (*Desulfovibrio*) were more abundant in the gut of wild horses than in wild asses, while Akkermansiaceae (*Akkermansia*), Lactobacillaceae (*Lactobacillus*), Bacteroidaceae (*Bacteroides*), and Spirochaetaceae (*Treponema*) were more abundant in wild asses (Figure 3).

### 3.3. Convergence in Functional Potential of Microbial Community

**Figure 4:**
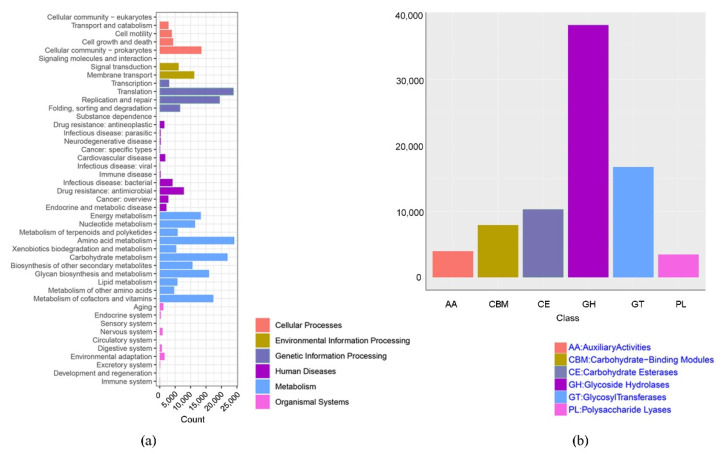
(**a**,**b**) Bar plots showing the relative abundance of genes associated with (**a**) the given KEGG pathways; and (**b**) CAZymes classes.

Functional profiles of metagenomic sequences were classified based on our newly assembled non-redundant gene catalogue (see Section 2.3). Approximately 2,581,290 genes were annotated using the KEGG database and 806,087 genes encoded known carbohydrate-active enzymes (CAZymes). The major functions predicted were related to metabolism (52.25%), genetic information processing (21.02%), cellular processes (9.41%), human diseases (8.3%), environmental information processing (7.01%) and organismal systems (1.92%) (Figure 4a). In the metabolism category, amino acid metabolism (17.69%), carbohydrate metabolism (16.1%), and metabolism of cofactors and vitamins (12.74%) were the most abundantly enriched pathways (Figure 4a). Most reported CAZyme genes were associated with the classes glycoside hydrolases (GH, 47.42%), glycosyltransferases (GT, 20.74%), and carbohydrate esterases (CE, 12.78, and fewer genes were associated with carbohydrate-binding modules (CBM, 9.83%), auxiliary activities (AA, 4.92%), and polysaccharide lyases (PL, 4.3%) (Figure 4b). In the two-level classification, we obtained 139 GH, 101 GT, 79 CBM, 16 CE, 26 PL, and 13 AA families, with GT2, GH2, GT41, GH109, and GT66 being the top five CAZymes (Figure S3).

**Figure 5:**
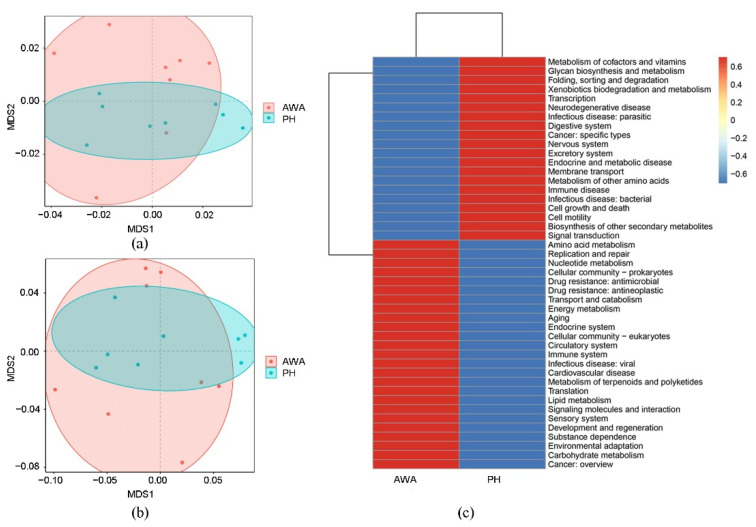
(**a**,**b**) NMDS plots showing the composition of genes related to (**a**) KEGG pathways (level 3); and (**b**) CAZymes, based on Bray–Curtis dissimilarities in Przewalski’s horses (PH) and Asiatic wild asses (AWA); (**c**) Heatmap showing the relative contribution of genes related to KEGG pathways (level 2).

**Figure 6:**
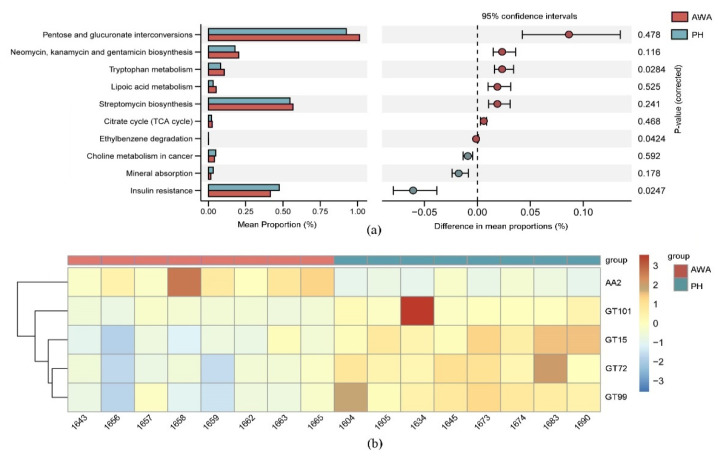
(**a**) The relative abundance of genes related to KEGG pathways (level 3) that were significantly different between Przewalski’s horses (PH) and Asiatic wild asses (AWA); (**b**) CAZymes that were significantly different between PH and AWA. The differences between groups were identified using STAMP and Metastats.

To investigate the difference between the abundances of functional genes and pathways between equid microbiomes, Bray Curtis distances were calculated using observation matrix tables containing information on KEGG and CAZy families. NMDS plots based on Bray–Curtis dissimilarities of genes related to the KEGG pathway (level 3) and CAZy families indicated that the microbial functional profile of wild horses was similar to that of wild asses (Figure 5a,b). A higher abundance of genes related to core metabolic functions (e.g., carbohydrate metabolism, energy metabolism, and amino acid metabolism) were enriched in wild asses (Figure 5c). However, only three pathways were found to be differentially abundant between wild horses and wild asses among the 362 KEGG pathways detected (level 3) (Figure 6a). Tryptophan metabolism and ethylbenzene degradation were positively associated with wild asses, whereas genes related to insulin resistance were more abundant in wild horses (Figure 6b). Among the carbohydrate-active enzymes, AA2 was significantly higher in wild asses, whereas four enzymes had higher proportions in wild horses, including GT101, GT15, GT72, and GT99.

## 4. Discussion

Although some datasets are available for the gut metagenomes of wild herbivores (e.g., *Camelus* [27] and yak [41]), little information is available on the metagenomes of wild equid microbiota. This study represents the first gut metagenomic characterization of sympatric reintroduced Przewalski’s horses and resident wild asses, allowing us to explore the relationship between the functional microbiome and the adaptive flexibility of wild equids under natural conditions. Our results demonstrated that the gut microbiota of wild horses and wild asses differed in species composition and tended to converge at species diversity/richness index and metabolism function levels, indicating functional equivalence in the metabolic functions of wild equids’ microbiomes.

Intestinal microbial diversity has been recognized as a new biomarker of health and metabolic performance in humans [42]. It seems intuitive that wild asses, with higher energy digestibility and more flexible diets, should harbor more diverse gut microbiota than wild horses. However, wild asses have slightly lower microbial diversity than reintroduced wild horses, which may be due to their smaller body size [43]. Microbial community diversity has been shown to decrease with host mass in herbivorous hosts [44,45]. Given the complexity of feeding behaviors under natural conditions, the relationship between diet diversity and gut microbiota diversity in the wild is still unexplored. Our results support the idea that microbial diversity cannot be oversimplified as a proxy for health or adaptation [46].

A significant difference was observed between the two hosts in microbial community composition based on weighted UniFrac distance, which considers both microbial composition and abundance. This result is consistent with those of previous studies on domestic horses and domestic donkeys [47]. However, the unweighted UniFrac method, which considers species presence/absence, showed no significant between-group variation in gut microbiome composition, indicating a large overlap of microbial species in the two hosts. In great apes, sympatric chimpanzees and gorillas have converged in terms of community composition [48]. Although horses and asses genetically represent two distinct lineages and harbor different lifestyles [49], it is possible for different host species occupying the same range to experience incidental or indirect contact through the environment, such as through soil, diet, or water resources, thereby providing a route for microbes to be transferred among individuals.

The gut microbiota of wild horses and wild asses differed at the taxonomic level in terms of relative abundance, while it was more convergent at the richness and functional levels, as indicated by the NMDS plot. One explanation for the incompatible pattern between taxonomic and functional profiles is that the relatively broad range of microbes that can serve similar digestive functions may reduce selection pressure and coexist in the gut environment. For example, *Ruminococcus*, which is enriched in wild horses, and *Bacteroides,* which is enriched in wild asses, are both known to play a role in fiber breakdown in the mammalian gut [50]. These results indicate that although Przewalski’s horses are less adaptive to the Gobi Desert environment than wild asses, the microbiome functional performance is sufficient for them to coexist with other equids. However, it should be noted that a previous study revealed that domestic donkey fecal microbiota had significantly higher bacterial and anaerobic fungal concentrations than the horse [47], which could enhance fiber degradation ability in the domestic donkey. The metagenomics approach is affected by systematic variability and may not represent the true absolute abundance of the species or genes [51]; therefore, further investigation is required.

We predicted that wild asses would be enriched in the microbiome and genes related to cellulose degradation and energy metabolism. Although the overall picture seems to be similar between equids, there is still some evidence concerning particular taxa and genes that supports our prediction. The abundance of Verrucomicrobia, mainly attributed to the genus *Akkermansia*, was significantly higher in wild asses. *Akkermansia* has also been found in previous studies in Przewalski’s horses [26], domestic donkeys [52], and Tibetan wild asses [53]. *A. glycanphila* and *A. muciniphila*, found in the current study, have been shown to degrade mucin, decrease bowel inflammation, and play important roles in maintaining the gut barrier [54,55]. Horses rely on the intestinal mucosa to maintain homeostasis, making them particularly vulnerable to clinical syndromes caused by ischemic mucosal injury [56]. A higher abundance of *Akkermansia* may help minimize friction damage in wild asses during the digestion of rough and fibrous meals, as well as chemical harm from toxic plant secondary metabolites.

The genes related to the pentose and glucuronate interconversions pathway (involved in carbohydrate metabolism), tryptophan metabolism (which promotes adaptive immune cell homeostasis), lipoic acid metabolism (which is related to mitochondrial metabolism), and the citrate cycle (a main energy source) were higher in abundance in wild asses than in wild horses. Of the total detected putative carbohydrate-active genes, AA2 (predominantly class II lignin-modifying peroxidases) expression was significantly increased in wild asses. This may be linked to the high efficiency of wild asses in digesting low-nutritional-value fiber [57]. While glycosyltransferases (GT101, GT15, GT72, and GT99) were detected at high percentages in wild horses, these enzymes are related to the establishment of natural glycosidic linkages and not associated with biomass deconstruction [58]. Our results imply that the differences in gut microbiota associated with certain metabolic pathways may be related to niche separation and the adaptive advantage of wild equids, which requires further research.

## 5. Conclusions

In conclusion, this study provides insights into the structure and function of the microbiome in sympatric equids. Our findings show that the microbiome in reintroduced horses and native Asiatic wild asses differ in taxonomic composition but are consistent in functional profile, indicating that the overall metabolic functional performance of gut microbes is not significantly associated with adaptation to arid environments in wild equids. However, we noted taxa and genes related to certain metabolic or functional pathways tend to be more abundant in wild asses, which requires further investigation. The major limitation of this study is the lack of biological information such as gender/age and the interaction with environmental factors such as diet. The interpretation of results is also influenced by the small sample size. Further investigation involving quantitative microbial profiling, diet investigation (e.g., stable isotope analysis, metabarcoding) and validation experiments (e.g., microbiome transplant) is required to elucidate the complexity of host-microbial interaction. Knowledge of the gut microbiomes of these two wild equid species may provide helpful insights for improving the performance of endangered, relocated species.

## Data Availability

All sequence data obtained in this study were deposited in the NCBI Sequence Read Archive (accession number: PRJNA814825).

## Supplementary Materials

The following supporting information can be downloaded at: https://www.mdpi.com/article/10.3390/microorganisms10061166/s1, Table S1: Details of metagenomic sequencing read statistics in each sample; Table S2: Assembly summary and predicted unigene statistics of metagenome in each sample; Figure S1: Rarefaction curves of observed species in fecal samples of Przewalski’s horses (PH) and Asiatic wild asses (AWA); Figure S2: Microbial community composition at (a) phylum and (b) genus level of Przewalski’s horses (PH) and Asiatic wild asses (AWA); Figure S3: Heat map shows distribution of CAZymes in gut microbiome of Przewalski’s horses (PH) and Asiatic wild asses (AWA).
