# Supplementary material for: Comparative Analysis of Microbiome Metagenomics in Reintroduced Wild Horses and Resident Asiatic Wild Asses in the Gobi Desert Steppe
Source: Microorganisms. 2022 Jun 7;10(6):1166. doi: 10.3390/microorganisms10061166 (PMC9229091; doi:10.3390/microorganisms10061166)
Supplement: Supplementary file 1 [file microorganisms-10-01166-s001.zip › microorganisms-1669990-supplementary.pdf]

Table S1 Details of metagenomic sequencing read statistics in each sample.

Sample	Host species	Total Reads	Clean Reads	Percentage	Clean bases	GC Content	%>Q20	%>Q30
X1604	PH	79138998	78506490	0.992	11845027134	0.4776	0.9857	0.9529
X1605	PH	86271658	84904396	0.9842	12813301089	0.4814	0.9853	0.9525
X1634	PH	86587912	85368404	0.9859	12880838153	0.4586	0.9853	0.9523
X1645	PH	81796664	80711160	0.9867	12178403671	0.4628	0.9847	0.9502
X1673	PH	88282058	87001818	0.9855	13129139132	0.4573	0.9845	0.9504
X1674	PH	84432202	83202886	0.9854	12553624350	0.473	0.9847	0.9506
X1683	PH	93989044	92691872	0.9862	13986062781	0.4711	0.9851	0.9517
X1690	PH	82985512	82394780	0.9929	12432462208	0.4737	0.9865	0.9549
X1643	AWA	79313198	78091258	0.9846	11782891091	0.5085	0.9847	0.9531
X1656	AWA	79716252	78295464	0.9822	11811954316	0.538	0.9819	0.9461
X1657	AWA	89195734	87755060	0.9838	13238904094	0.5034	0.982	0.9457
X1658	AWA	84097924	82538608	0.9815	12453082080	0.5022	0.9802	0.9413
X1659	AWA	83245146	81576992	0.98	12305278054	0.4951	0.9803	0.9411
X1662	AWA	84097220	82525996	0.9813	12446926697	0.4836	0.9824	0.9468
X1663	AWA	83422354	81394668	0.9757	12267789692	0.474	0.9809	0.9429
X1665	AWA	83564246	82483074	0.9871	12436956767	0.4784	0.9829	0.9478

Note: Total and clean reads indicate pair-end reads counts before and after quality control. Clean base refers to the number of bases in clean data. ≥Q30% refer to the percentage of bases with quality scores greater or equal to 30. PH= Przewalski’s horse. AWA= Asiatic wild asse.

Table S2 Assembly summary and predicted unigene statistics of metagenome in each sample.

Sample	Host species	Num_contigs	Total_length (bp)	Min_length	Max_length	Average_length	N50	Number of unigenes
X1604	PH	685,878	780,341,404	500	355,253	1,137.70	1,199	3098568
X1605	PH	782,499	775,588,316	500	301,782	991.2	982	4168394
X1634	PH	660,327	706,101,222	500	616,284	1,069.30	1,075	3531223
X1645	PH	637,926	700,330,517	500	333,554	1,097.80	1,119	3871114
X1673	PH	769,154	777,144,656	500	421,439	1,010.40	1,005	3816935
X1674	PH	734,085	752,554,983	500	375,595	1,025.20	1,023	4282943
X1683	PH	763,980	792,222,554	500	127,262	1,037	1,048	3834544
X1690	PH	737,444	780,477,772	500	285,692	1,058.40	1,062	3756167
X1643	AWA	826,975	878,820,006	500	247,234	1,062.70	1,086	3581794
X1656	AWA	650,396	761,843,907	500	361,402	1,171.40	1,253	3271950
X1657	AWA	789,777	881,357,651	500	281,438	1,116	1,162	4116908
X1658	AWA	692,537	799,178,451	500	374,559	1,154	1,202	4100099
X1659	AWA	723,437	799,583,128	500	331,885	1,105.30	1,137	4069132
X1662	AWA	709,165	797,595,808	500	154,257	1,124.70	1,157	3664719
X1663	AWA	756,896	805,724,983	500	188,002	1,064.50	1,078	4084772
X1665	AWA	755,046	809,607,841	500	213,916	1,072.30	1,098	3680232

Note: PH= Przewalski’s horse. AWA= Asiatic wild asse.

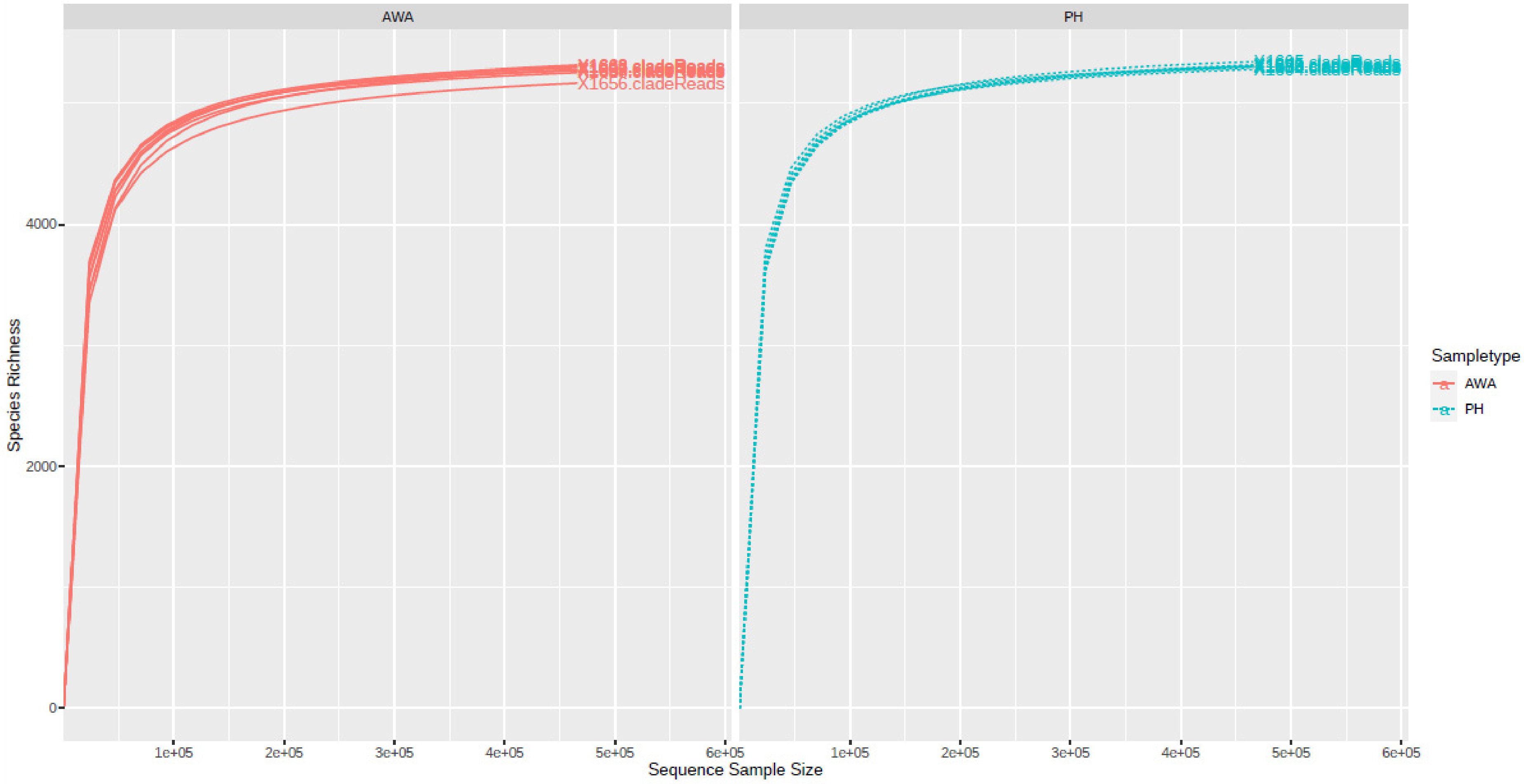

Figure S1 Rarefaction curves of observed species in fecal samples of Przewalski's horses (PH) and Asiatic wild asses (AWA).

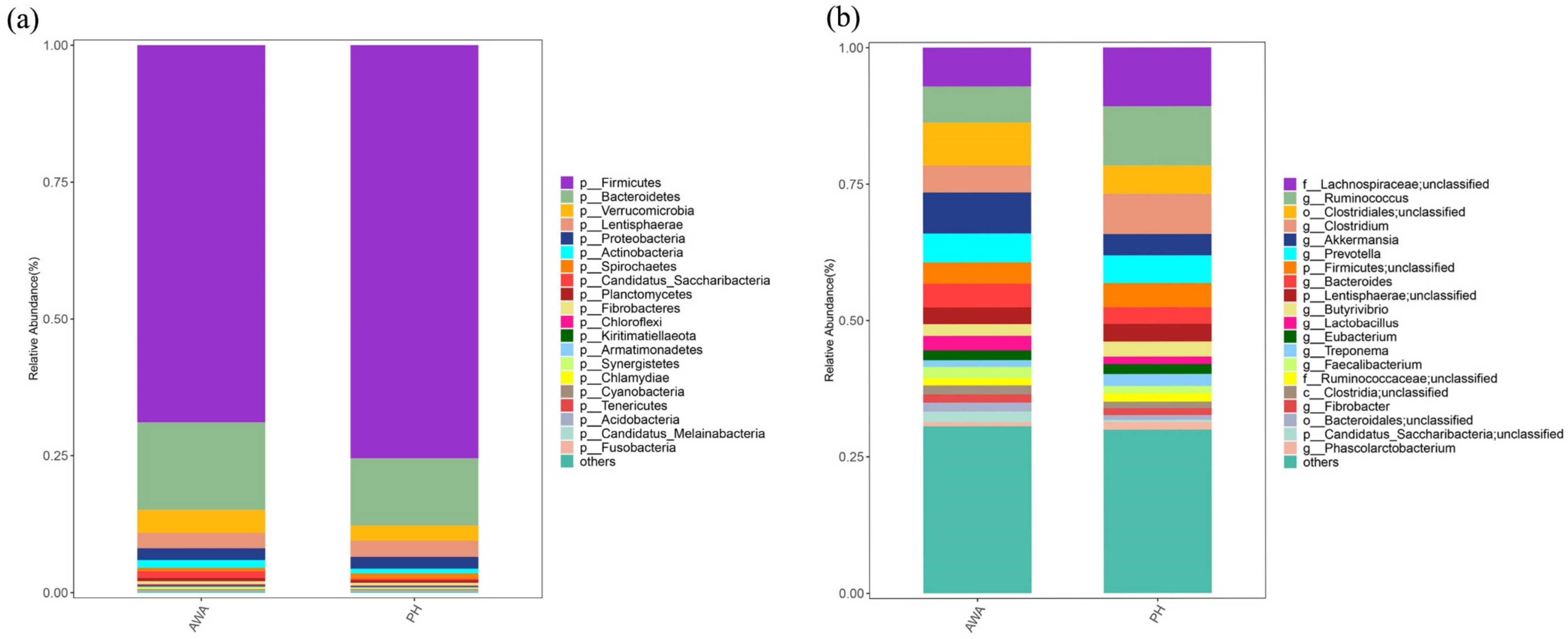

Figure S2. Microbial community composition at (a) phylum and (b) genus level of Przewalski's horses (PH) and Asiatic wild asses (AWA). The x-axis represents groups and the y-axis represents relative abundance. The taxa that have relative abundance less than 0.01 were combined as "others".

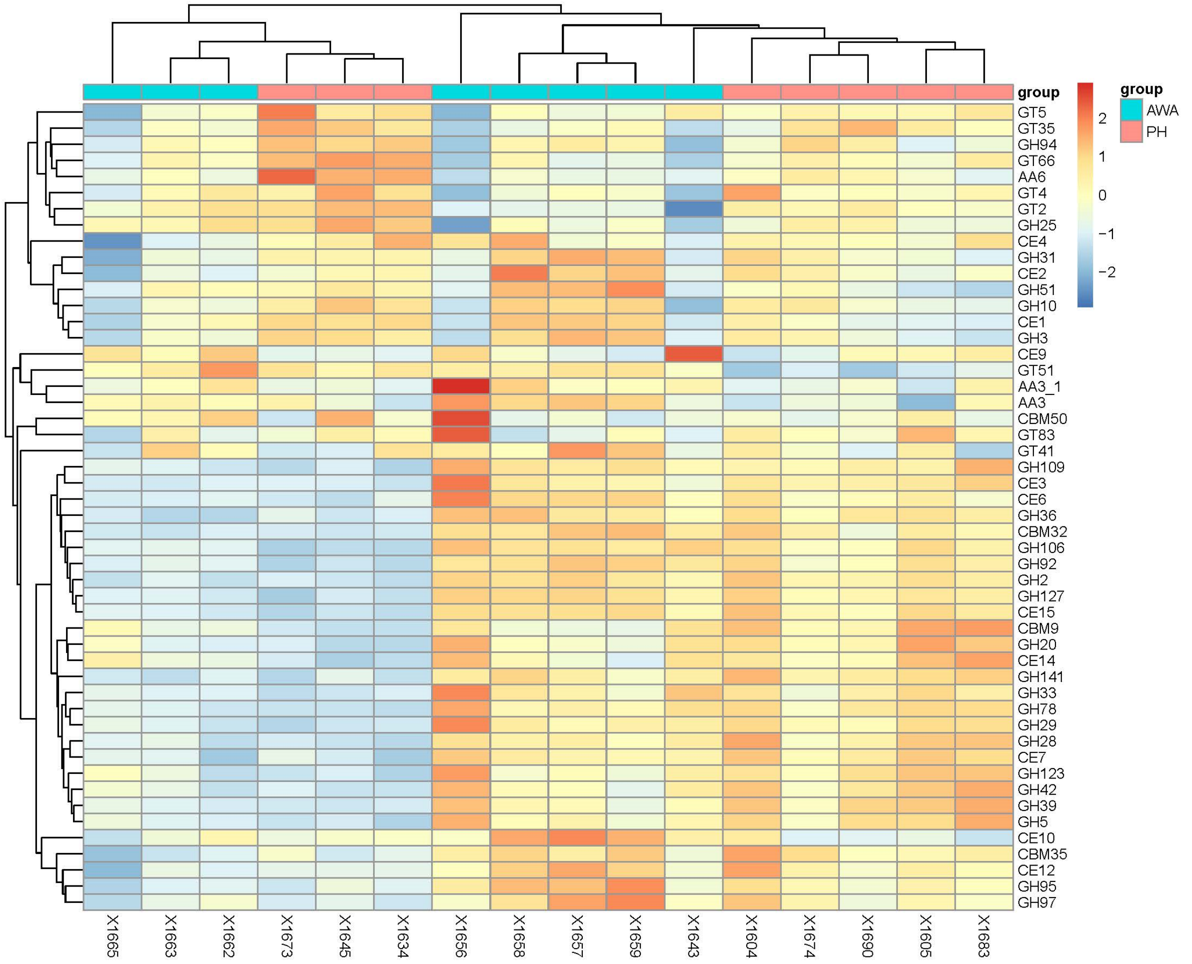

Figure S3 Heat map shows the distribution of CAZymes in the gut microbiome of Przewalski's horses (PH) and Asiatic wild asses (AWA).
